# Jamestown Canyon Virus Disease: An Analytic Review of Human Cases Reported from 1982 Through 2022

**DOI:** 10.3390/v18020271

**Published:** 2026-02-23

**Authors:** Stephen F. Johnson, Karin E. Peterson

**Affiliations:** Laboratory of Neurological Infections and Immunity, Rocky Mountain Laboratories, National Institute of Allergy and Infectious Diseases, Hamilton, MT 59840, USA; stephen.johnson4@nih.gov

**Keywords:** Jamestown Canyon, arbovirus, encephalitis, diagnosis, nervous system

## Abstract

Reports of acute Jamestown Canyon Virus (JCV) cases have increased markedly over the last 15 years, associated with improved diagnostic testing protocols. Analysis of these cases and the criteria used for their diagnosis could benefit clinicians encountering this under-recognized disease. In the current study, we analyzed all published reports of acute human JCV infections in North America from the first in 1982 through 2022, including retrospective studies. A total of 50 reports with 416 cases of JCV were found. The primary illness associated with JCV infection involved the nervous system. Of reported encephalitis cases, the fatality rate was 2.4 percent in hospitalized patients. Of the cases with detailed patient outcome information, approximately 40 percent had prolonged hospitalization and/or long-term neurological sequelae. Although case incidence has increased over the last few decades, the overall time from admission/clinical onset to testing for JCV has not substantially changed from the 1980s. Confounding factors such as being immunocompromised, as well as previous or concurrent infections, were associated with a greater risk of more severe outcomes. Thus, the complexity of JCV infection with other conditions may impact the overall outcomes of JCV encephalitis.

## 1. Introduction

Neuroinvasive infections are a serious threat to patients but are difficult to diagnose. Early manifestations can include headache, fever, poor ability to concentrate, malaise, and dizziness, which are nonspecific and similar to other febrile illnesses. Clinical symptoms characteristic of acute encephalitis syndrome (AES) may present later, limiting the timeframe for efficient evaluation and diagnosis to prevent further damage to the nervous system [1,2]. Indeed, in approximately 50% of encephalitic cases, no etiology for meningoencephalitis is confirmed [1,3], which may be due to delays in, or lack of, diagnostic testing [4]. Multiple efforts have been made to improve diagnosis over the last 60 years, including insightful reviews, clinical-pathological teaching conferences, and better diagnostics [1,2,4,5,6,7,8,9]. Analyzing the reported cases for different encephalitic viruses may provide clues to help understand the specific parameters of diagnosis and provide guidance in diagnosis. In the current manuscript, we analyzed the reported cases for Jamestown Canyon Virus (JCV), a virus endemic to North America and an important cause of viral encephalitis [10,11,12,13].

JCV was first isolated in 1961 from mosquitoes isolated from Jamestown Canyon, Colorado [14]. Serological testing identified JCV as a member of the California Serogroup (CSG) of Orthobunyaviruses, based on serological cross-reactivity with California encephalitis virus (CEV) [14,15]. Other members of this serogroup include La Crosse virus (LACV), snowshoe hare virus (SSHV), trivittatus virus (TVTV), keystone virus (KEYV), and Inkoo virus (INKV). Seroprevalence of JCV and virus detection in mosquitoes indicate that JCV is widespread in North America, being found in Canada, Greenland, and the United States of America (USA) [10,16,17,18,19,20,21]. Seroprevalence studies in humans show a wide range of human exposure to JCV, with positive neutralizing antibodies (NAbs) detected in 4% of the population in one study to 42% of samples from the upper peninsula of Michigan [10,17,20,21,22]. In Nova Scotia, seropositivity in human samples ranged from 15% to 49%, with the highest seropositivity in a rural health care district [23]. These data indicate that JCV infection in humans occurs frequently and may be higher in rural settings than in urban environments.

**Figure 1:**
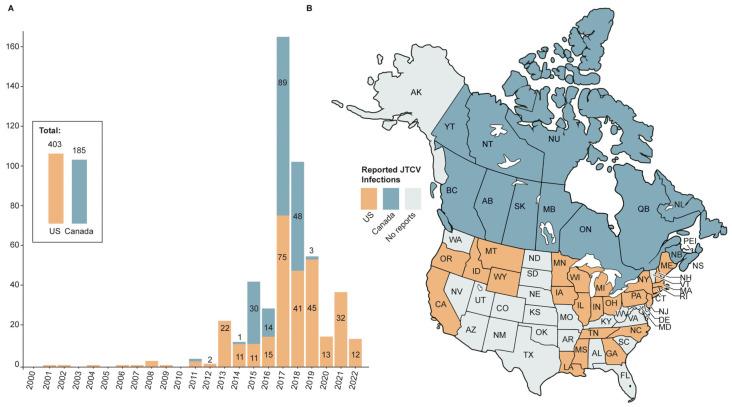
Number and location of JCV cases in Canada and the USA from 2000 to 2022. (**A**) Number of reported cases of JCV illnesses in the USA (orange) and Canada (blue) per year per CDC and CNML reports (described in Table S1). (**B**) States and provinces with at least one case of JCV illness between 2000 and 2022. All Canadian provinces reported at least one case within this time frame.

Despite JCV seropositivity being widespread in North America, the actual number of confirmed human JCV encephalitis cases was relatively low until the early 2000s. The first reported acute human disease caused by JCV was not diagnosed until 1980 [24]. Speculation that JCV encephalitis was an emerging human disease was raised in a case series of 12 acute human JCV infections reported in 1983 in Ontario, Canada and upstate New York, USA [10]. A subsequent retrospective study of acute encephalitis of uncertain etiology in upstate New York detected 31 additional cases associated with positive plaque reduction neutralization test (PRNT) for JCV [25]. However, during the next 30 years, few additional human JCV infections were reported [10,22,26,27]. Screening with a virus-specific JCV IgM-capture-enzyme-linked immunosorbent assay (JCV IgM) was employed by the US Center for Disease Control (CDC) on all cases of suspected neuroinvasive arbovirus infection in 2013 [27]. The Canadian National Microbiology Laboratory (CNML) added JCV IgM screening to the samples that they checked for West Nile virus (WNV) infection in 2015. These new screening methods resulted in an increase in detectable JCV cases in the United States and Canada (Figure 1A) suggesting that these cases were previously underreported.

To better understand JCV cases, we examined historical and current case reports of JCV encephalitis. We compare here the clinical symptoms and signs, method of diagnosis, confounding factors, and patient outcomes in order to determine commonalities or unique features that might aid the clinician with understanding the full breadth of clinical manifestations of JCV cases.

## 2. Methods

### 2.1. Analysis of the Literature and Determination of Cases for Inclusion as JCV

For this study, we searched the annual *Morbidity and Mortality Weekly Report (MMWR)* of *West Nile Virus and Other Arbovirus Diseases*, the Public Health Agency of Canada’s *West Nile Virus and Other Mosquito-Borne Diseases Surveillance Report: Annual Edition*, as well as completing a PubMed search for case reports on Jamestown Canyon viruses. All included cases are reported in Table S1. *ArboNET* data (Historic Data (2011–2024) | Jamestown Canyon Virus | CDC) was used only to clarify the origin or diagnostic classification of some cases published in the annual *MMWR*. We counted the cases in the *MMWR* annual reports and did not include *ArboNET* results separately in our totals. We compared location, year, as well as ages and sex when available, for cases reported by the Centers for Disease Control (CDC), medical journals, and CNML to remove duplicated entries.

Our compilation of 44 references from PubMed is complete for the years 1971 through 2022, including some pre-1982 acute cases of unknown etiology tested retrospectively from serum stored in the Upstate New York Laboratory (Table S1). Consistent numbers were difficult to establish due to incomplete reporting from the Canadian National Microbiology Laboratory (CNML), which disclosed numbers of positive CSG tests from 2013 to 2018 but stopped reporting in 2019 because “CSG virus infections are not nationally notifiable diseases in Canada, so there is no formal surveillance system in place to monitor, track, and report cases” [28]. References are provided for all cases within the tables.

### 2.2. Analysis of Case Reports for Lumbar Puncture, Sample Sendoff, Awareness Interval Time, and Lab Results

When authors of a JCV report determined the first day of a patient’s illness and the reported time of cerebral spinal fluid collection, we employed that time in weeks as the CSF time interval (CTI). Otherwise, we employed the day of first contact of the patient with a clinician as the best marker to indicate CTI. Of the JCV cases reported, information for CTI was available for 34 cases. CTI for one patient (23, case 6) was more than 4.6 standard deviations above the mean, and onset of illness was unclear from the narrative, as the patient was only seen on a single occasion and then failed to follow up. These factors seemed sufficient to consider her an outlier and to exclude her from CTI analysis. Another case with prolonged CTI [5] associated with rituximab treatment was excluded due to the time of illness onset being unclear.

Awareness Time Interval (ATI) was calculated as the time from illness onset to the day of the first specimen draw that later implicated JCV as a probable or confirmed cause of the patient’s illness. This interval could be estimated for 41 of 49 cases. The actual time for laboratory JCV results from sample sendoff to laboratory results reported back to the clinicians could be determined for only 10 cases.

### 2.3. Analysis of Cases of Meeting CDC Criteria for Confirmation as JCV Positive

The CDC laboratory criteria for diagnosis of arboviral encephalitis include (i) “isolation of virus from, or demonstration of specific viral antigen or nucleic acid in, tissue, blood, CSF, or other body fluid, OR” (ii) “fourfold or greater change in virus-specific quantitative antibody titers in paired sera, OR” (iii) “virus-specific IgM antibodies in serum with confirmatory virus-specific neutralizing antibodies in the same or a later specimen, OR” (iv) “virus-specific IgM antibodies in CSF or serum” (https://ndc.services.cdc.gov/case-definitions/arboviral-diseases-neuroinvasive-and-non-neuroinvasive-2015/, accessed on 10 February 2026). Cases with sufficient information were analyzed on whether they met any of the above criteria as confirmation of being positive for JCV. We further devised the following definitions for probable reports of JCV with subcategories of P1-P4 for defining components that did not allow for full CDC criteria. These were P1 = inadequate clinical detail for neuroinvasive and non-neuroinvasive disease or to exclude a more likely explanation of symptoms or no specification of type of clinician, P2 = JCV IgM positive, but PRNT unchanging, P3 = inadequate testing for other probable viral causes, and P4 = inadequate testing by PRNT or IgM to exclude probable viral cross-reactive antibodies.

### 2.4. Analysis of Cases for Acute Encephalitis Syndrome (AES)

We examined the 49 detailed reports for AES criteria based on Venkatesan et al. [2]. The major criterion required is “Neurological dysfunction manifesting with altered mental status for more than 24 h without an alternative cause”. Minor criteria include: (a): Documented fever ≥ 38 °C (100.4 °F) within the 72 h before or after presentation”, (b) “generalized or partial seizures not fully attributable to a preexisting seizure disorder”, (c) “new onset of focal neurologic findings”, (d) “CSF WBC count ≥ 5/cubic mm^3^, (e) abnormality of brain parenchyma on neuroimaging” suggestive of encephalitis that is either new from prior studies or appears acute in onset, and (f) abnormality on electroencephalography that is consistent with encephalitis and not attributable to another cause.

## 3. Results

### 3.1. Human Case Reports of JCV

A total of 598 cases of persons with acute JCV infections have been reported since 1971 in North America (Table S1) [4,7,10,12,19,22,24,25,29,30,31,32,33,34,35,36,37,38,39,40,41,42,43,44,45,46,47,48,49,50,51,52,53,54,55,56,57,58,59,60,61,62,63,64,65,66,67,68]. Surveillance efforts by the US Center for Disease Control (CDC) or the Canadian National Microbiology Laboratory (CNML) account for at least 83 percent (498/598) of reported JCV infections, and those two reference laboratories assisted with the diagnosis of most of the remaining cases.

Clinical categories of diagnosis were available in a total of 416 JCV cases, including neurological and non-neurological disease (Table S1). Disease severity ranged from nonspecific fever to severe long-term impairment or death (Table S1). The major diagnosis in 291 of 416 (70.0%) of JCV infections involved the nervous system. The most severe clinical diagnosis was encephalitis (including acute encephalitis syndrome (AES) and meningoencephalitis), which occurred in 154 of 416 (37%) of the patients whose diagnosis was reported (Table S1). JCV meningitis occurred in 78 of 416 (18.8%) of patients. Clinical signs of JCV meningitis differed from those of encephalitis in that patients retained normal cognitive processing, levels of consciousness, absence of seizures, and a faster recovery time compared to encephalitis cases. Acute flaccid paralysis was reported for 12 cases (2.9%). An additional 61 patients were diagnosed as simply “neuroinvasive disease”. The 30% of symptomatic patients who were positive for JCV infection but not neuroinvasive disease had various defining illnesses, including one patient with pneumonia [10] and another with sepsis [58]. Non-neuroinvasive disease was observed in 125 of the 598 JCV infections (Table S1) [10,17,20,22,23]. Of these non-neurological cases, there are few details for long-term analysis. For example, seven cases were reported as “febrile illness without neurological involvement” with no further information [27].

### 3.2. JCV Cases with Detailed Analysis for Further Evaluation

Of the 416 cases of JCV infection, 49 individual case reports had sufficient information to compare their assessment with CDC arbovirus disease case definitions, clinical process of JCV diagnosis, and outcomes (Table 1, Table 2 and Table 3). Of the detailed individual JCV cases, 30 of the 49 cases (61%) were diagnosed with encephalitis (Table 1) per CDC definition (see Section 2). Acute encephalitis syndrome (AES) diagnosis, which requires a single major and three minor criteria as described in the methods, were found in 12 of the 30 encephalitis cases (40%). In 11 of the 30 encephalitic cases, brain biopsy or brain MRI were used to verify the diagnosis of encephalitis (Table 1). Four of the 49 cases were classified as non-neuroinvasive cases. One patient was reported with “viral illness”, another with “pneumonia,” and a third with “possible dengue” [10,22,50]. The fourth was a patients with sepsis [60]. Thus, of the 49 cases with detailed analysis, over half were diagnosed as encephalitis.

### 3.3. Probable and Confirmed Neuroinvasive Arbovirus Infection

We next analyzed the detailed case reports to determine whether they met probable or confirmed criteria for JCV based on CDC definition. Of the 49 cases, 28 (57%) met the “confirmed” case definitions (Table 2, column CR). The remaining 21 cases were classified as not fully confirmed (probable) for one or more of 4 reasons: (P1) insufficient narrative detail about the type of clinical involvement to support neuroinvasive or non-neuroinvasive infection or to exclude a more likely explanation of symptoms; (P2) JCV titer high but not increasing; (P3) insufficient testing for other probable viral causes, or (P4) insufficient rise in PRNT or IgM levels to exclude viral cross-reactive antibodies. Three cases had multiple reasons for rating them as “probable” rather than “confirmed.” No cases were defined as only P1, four cases were defined as P2, two cases for P3 and 12 cases for P4. Thus, the majority of cases met the CDC criteria for JCV encephalitis. Of the cases that did not, the general reason was due to a lack of sufficient rise in PRNT or IgM levels as an indication of active infection.

### 3.4. Timeline for Analysis of JCV as a Causative Agent

**Figure 2:**
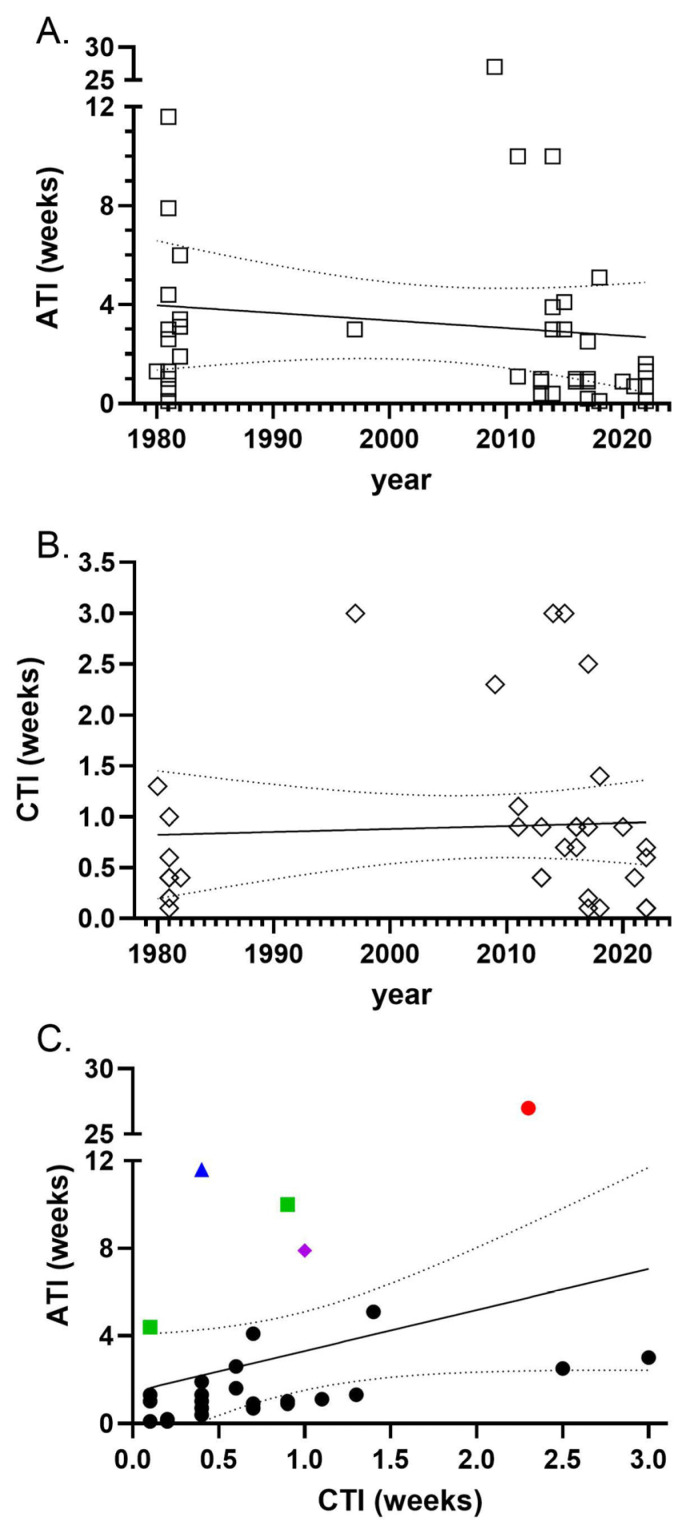
Awareness interval time (ATI) and CSF time interval (CTI) for JCV cases. (**A**) Time in weeks from report of initial symptoms until samples were sent off for analysis for JCV infection (IgM or neutralizing antibodies). ATI was determined from 41 cases that contained this information and plotted versus the case year. Linear regression analysis found an R squared value of 0.012 and no significant deviation from 0. (**B**) The time from report of initial symptoms to lumbar puncture being taken (CTI) for analysis was determined for 34 cases and plotted versus case year. Linear regression analysis found an R squared value of 0.002 and no significant deviation from 0. (**C**) Comparison of ATI and CTI for 32 cases with both information provided. Colored symbols are outliers and are explained under Section 3.4. A linear regression indicated an R value of 0.10 and no significant deviation from 0, indicating that the two values were not directly linked.

To gain information on the time frame for diagnosis, we computed an awareness time interval (ATI) from the reported day of illness to when samples were sent out to test for JCV infection. ATI could be measured in 41 cases with a median ATI of 1.3 weeks. From 1981 to 2022, the time needed to recognize possible JCV infection did not significantly change (Figure 2A). Another time interval, from clinical onset to when cerebral spinal fluid was taken for diagnostic testing, was recently shown as a critical factor in length of time to diagnosis for HSV-1 [69]. We next calculated this measurement (CSF time interval, CTI), which was available for 36 JCV cases. From 1980 to 2022, CTI increased slightly by 1 day (Figure 2B). For the 6 cases whose CTI delays were significantly longer than the trend line, the explanations were slow clinician recognition of acute encephalitis syndrome [70], patient delay before contacting a clinician [10,27], and slow disease progression [27]. Both the CTI and ATI were available for 32 cases (Figure 2C). Shorter CTI was generally associated with shorter ATI. Outliers of ATI were associated with delayed use of serum IgM for diagnosis (cases 7 and 4, Figure 2C green squares), slow rise in IgG and IgM (case 6, blue triangle), or an immune-compromised heart transplant patient who had both slow rise and delayed serum draws for IgG and IgM analysis (purple diamond) [34]. The largest outlier (red circle) was associated with persistent IgM from previous WNV infection and delayed serum draws of convalescent IgG testing [42]. Thus, there was no offset in timelines between general testing for encephalitic viruses via CSF collection (CTI) and specific testing for JCV (ATI). However, the time frame for each has not decreased over time but has remained relatively flat, indicating no improvement in earlier detection of JCV cases.

**Table 1:** Diagnosis and outcomes of cases with detailed information.

Year ^a^	Age, Sex ^b^	Diag ^c^	Prod ^d^	AES ^e^ Maj/min	Outcome ^f^	Ref, Case
*1980*	*8 F*	*E*	*Yes*	*1/5*	*Hospitalized 27 days, no sequelae at 15 months*	[10,24]*, 1*
1981	29 M	M	Yes	0/2	Recovered 10 days later	[10], 2
1981	39 F	M	Yes	0/1	**Cognitively impaired for 2–4 months**	[10], 3
1981	18 M	NN	Yes	0/0	Discharged day 4	[10], 4
1981	31 M	M	Yes	0/2	Discharge at 6 days, no sequelae at 16 months	[10], 5
1981	31 M	M	Yes	0/2	Discharge at 3 days, no residua at 12 months	[10], 6
1981	87 M	E	Yes	1/1	NR	[10], 7
1981	34 M	P	Yes	0/1	NR	[10], 8
1981	21 F	M	Yes	0/2	Discharge at 3 days, no residua at 1 year	[10], 9
1981	22 F	NR	Yes	0	NR	[10], 12
*1982*	*44 F*	*E*	*NR*	*1/2*	***Died***	[10], *10*
1982	24 M	NN	NR	0/1	NR	[10], 11
1982	52 M	E	Yes	1/4	NR	[22], 1
1982	C M	NN	Yes	0/0	NR	[22], 2
1983	14 M	M	NR	0/0	NR	[22], 3
1984	11 M	M	NR	NR	NR	[70]
*1997*	*20 F*	*E*	*Yes*	*1/1*	***2 months in hospital, 6 months LTC, severely disabled***	[26]
2001	T M	M	Yes	0/2	Full recovery	[71]
2009	51 M	E	None	0/2	Recovered by 6 months. Follow up confirmed E.	[42]
2011	53 M	E	Yes	1/2	**Hospitalized 28 days. Expressive aphasia persistent at 6 month follow up**	[58], 4
2011	48 M	E	Yes	1/4	Full recovery	[58], 2
2013	66 M	E	Yes	1/4	**Unable to resume work due to poor memory, ataxia, and depression**	[58], 1
2013	65 M	E	Yes	1/3	**Prolonged sequelae, brain atrophy**	[58], 3
2016	70 M	E	Yes	1/2	Hospitalized 5 weeks, but full recovery	[58], 5
*2011–16*	80 F	*E*	*NR*	*1/2*	***Died***	[12]
*2013*	*57 M*	*E*	*Yes*	*1/3*	***1 month in hospital, poor memory for several months***	[50]*, 1*
2014	65 M	E	Yes	1/3	**Prolonged symptoms, brain biopsy was misleading**	[50], 2
2014	31 M	NN	Yes	0/0	Recovered after 10 weeks	[50], 3
2014	63 M	NN	Yes	0/1	Recovered by 2 months	[50], 4
2015	36 M	E	Yes	0/2	**Brain MRI verified E, symptoms lasted >1 year**	[50], 5
2016	68 F	NN	Yes	0/0	NR	[50], 6
*2016*	*57 M*	*E*	*Yes*	*1/1*	*Ongoing fatigue but working after 2 months*	[50]*, 7*
2017	28 M	NN	Yes	1/0	**Dull headaches for >6 months**	[50], 8
2017	40 M	E	None	1/2	Retrograde amnesia precluded return to work for >1 month	[50], 9
2014	62 M	S	Yes	1/1	Hospitalized 5 days, symptoms better after 2 months	[60]
*2015*	*73 M*	*E*	*Yes*	*1/2*	***Post-encephalitis dementia***	[68]
2016	31 M	E	Yes	1/3	**EEG improved on ribavirin, but patient died of sepsis. Biopsy confirmed E**	[62]
2017	26 M	E	Yes	0/3	Brain MRI verified E, 5 days in hospital, fully recovered	[67]
2017	Ol M	E	Yes	1/2	**In hospital 21 days; deficits resolved by 4 months post-onset**	[46]
*2017*	*56 M*	*E*	*Yes*	*1/3*	***Brain biopsy verified E. Death and necropsy***	[4]
2018	10 F	E	Yes	0/3	Brain MRI verified E. 4 days in hospital, was well at 2.5 weeks	[55,56]
*2018*	*48 F*	*E*	*Yes*	*1/1*	*3 hospitalizations within 53 days, then rehabilitation. Normal activities and cognitive function recovered*	[34]
2020	59 M	E	Yes	1/2	**Brain MRI verified E. Died on hospital day 21**	[66]
2021	59 M	E	Yes	1/3	**Brain MRI consistent with E. Cognitive deficits lingered and required family supervision**	[52]
2022	79 M	E	Yes	0/2	Brain biopsy and brain MRI verified E. Improved by day 14 in hospital	[59]
2022	57 F	E	Yes	1/3	JTCV precipitated MOGAD. Initial cognitive impairment, but back to baseline at 2 months post	[32]
*2022*	*36 M*	*M*	*Yes*	*0/3*	*Headache, neck stiffness and pain improved by day 6*	[72]
*2018*	*4 F*	*E*	*None*	*?/3*	***Stable, but significant neurologic impairment***	[61]
*2022*	*43 M*	*E*	*Yes*	*1/3*	***6 months after hospital discharge returned to work full-time***	[49]

(a) year case diagnosed, for series, year first case was diagnosed. Diagnostics for these cases are presented in Table 2. Cases in italics are further described in Table 3. (b) age in years and sex, M: male, F: female, C: college age, Ol: older mid-50s, T: teen. (c) Diagnosis of disease: AFP: acute flaccid paralysis, E: encephalitis, M: meningitis, ME: meningoencephalitis, NI: neuroinvasive, NN: not neuroinvasive, NR: not reported, P: pneumonia, S: sepsis. (d) Prod: Prodrome. Whether the patient reported initial symptoms of headache, confusion, anxiety, and irritability. (e) AES: acute encephalitis syndrome. Criteria based on Venkatesan et al. Table 1 [2]. Major criteria: Neurological dysfunction manifesting with altered mental status for more than 24 h without an alternative cause. Minor Criteria: (a) Documented fever ≥ 38 °C (100.4 °F) within the 72 h before or after presentation (b) generalized or partial seizures not fully attributable to a preexisting seizure disorder (c) new onset of focal neurologic findings (d) CSF WBC count ≥ 5/cubic mm^3^ (e) abnormality of brain parenchyma on neuroimaging suggestive of encephalitis that is either new from prior studies or appears acute in onset (f) abnormality on electroencephalography that is consistent with encephalitis and not attributable to another cause. (f) Outcomes as provided in the study. LTC: long-term care, NR: not reported or limited clinical information. Cases in bold indicate long-term impact or death.

**Table 2:** Symptomatology and diagnostic tests of cases in Table 1.

Year, Case ^a^	Symptoms ^b^	LP ^c^	Tests ^d^	Serology Tests: + if Positive ^e^	SO ^f^	CSG Viruses Tested ^g^	SR ^h^	JTCV Results ^i^	CR ^j^	Ref
1980, 1	8-day prodrome with fever+, then seizures and coma	1.3	CT, LP+, EEG+	EEE, SLE, WEE, but HSV+	1.3	LACV, CEV, SSHV, KEYV, TVTV, TAHV, SANV	NR	IgM +, PRNT 4-fold rise in paired-sera	C	[24]
1981, 2	Prodrome, HA, shaking chills+	<0.4	LP+	EEE, HSV, WEE, SLE, POW	1.3	LACV, KEYV, SSHV	NR	IgM not tested, PRNT 8-fold rise	C	[10]
1981, 3	Prodrome, fever+, stiff neck	No LP	NR	EEE, HSV WEE, SLE, POW, mmp, meas	3.0	LACV, KEYV, SSHV	NR	IgM+ PRNT 16-fold rise	C	[10]
1981, 4	Prodrome, HA, stiff neck	1.0	LP	EEE, WEE, SLE, HSV, POW, mmp, meas	7.9	LACV, KEYV, SSHV	NR	PRNT stable and 8-fold higher, IgM+	P2	[10]
1981, 5	HA, fever+, abnormal CSF	0.2	LP+	EEE, WEE, SLE, HSV, POW, mmp, meas	0.1	LACV, KEYV, SSHV	NR	IgM+, PRNT 8-fold rise	P4	[10]
1981, 6	Prodrome, HA, fever+	0.4	LP+	EEE, WEE, SLE, HSV, POW, mmp, meas	11.6	LACV, SSHV, KEYV	NR	Delayed IgM and PRNT 4-fold rise	C	[10]
1981, 7	Prodrome, coma+	0.1	LP+	EEE, WEE, SLE, HSV, POW, mmp, meas	4.4	LACV, SSHV, KEYV	NR	CF 2-fold rise, IgM+, PRNT 1:81,920	P2	[10]
1981, 8	HA, fever+, cough, stiff neck, HA	No LP	Chest X-Ray+	EEE, WEE, SLE, HSV, POW, mmp, meas	1.0	LACV, SSHV, KEYV	NR	HI 8-fold rise, IgM+++, then dropped to 0, PRNT 1:5120	C	[10]
1981, 9	Prodrome, HA, fever+	0.6	LP+	EEE, WEE, SLE, HSV, POW, mmp, meas	2.6	LACV, SSHV, KEYV	NR	HI 4-fold rise, IgM+ PRNT 1:640	C	[10]
1981, 12	HA, stiff neck	No LP	NR	EEE, WEE, SLE, HSV, POW, mmp, meas	0.6	LACV, SSHV, KEYV	NR	IgM+, PRNT 1:320	P2	[10]
1982, 10	Seizures+, coma+, LP+	0.4	NR	EEE, WEE, SLE, POW, mmp, meas, HSV+ 16-fold rise	1.9	LACV, SSHV, KEYV	NR	IgM+++ and JCV PRNT 1:80 through-out	P2	[10]
1982, 11	HA, fever+	No LP	NR	EEE, WEE, SLE, HSV, POW, mmp, meas	3.1	LACV, SSHV, KEYV	NR	IgM negative to ++ and PRNT 4-fold rise	C	[10]
1982, 1	HA, fever+, coma+, paralysis+, seizures+	NR	NR	HSV and other CNS pathogens	3.4	LAC, TVT	NR	>2-fold PRNT titer rise, IgM NR	P4	[22]
1982, 2	Vesiculopapular rash	NR	NR	HSV	NR	LAC, TVT	NR	16-fold PRNT titer rise, IgM NR	C	[22]
1983, 3	Meningeal signs	NR	NR	HSV	6.0	LAC, TVT	NR	4-fold PRNT titer rise, IgM NR	C	[22]
1984	Aseptic meningitis	NR	NR	NR	NR	CSG viruses, but members NR	None	Highest titers of HI, CF, PRNT, IgM+	P1-P4	[70]
1997	Prodrome, confusion+, HA, fever+	3.0	LP+, CT, EEG, MRI+	EEE, WEE, SLE, POW, HSV, CMV, EBV, HIV	3.0	LAC	NR	Initial CSF RT-PCR+, brain biopsy RT-PCR+	C	[26]
2001	HA, fever+, diplopia+	NR	NR	NR	NR	NR	NR	IgM+ by IFA, PRNT+	P1, P3, P4	[71]
2009	HA, fever+, left side numbness+, mosquitoes	2.3	CT, MRI, LP, Carotid ultra-sound	SLE. WNV (IgM+, but PRNT 1:320 unchanged)	1.0	LACV	NR	IgM+, 4-fold PRNT rise	C	[42]
2011, 4	Prodrome, fever+, rash, confused+, HA	1.1	LP	WNV, POW, EEE, WEE, Rickettsia, Borrelia, Ana-plasma+, but stable	1.1	SSHV + but IgM NR	NR	CSF IgM+ and PRNT increased 16-fold, serum IgM+ and PRNT increased	C	[58]
2011, 2	Prodrome, HA, ataxia+, confusion+	0.9	CT, LP+, EEG+, MRI+	HSV, VZV	1.0	SSHV	NR	IgM+ serum, PRNT rose 0 to 1:80	C	[58]
2013, 1	Prodrome, fever+, confusion+	0.4	EEG+, MRI+	Crypto, HSV, VZV, >12 other micro-biology	0.4	SSHV CSF PRNT and IgM negative	NR	Serum PRNT IgM+ 1:40 then 1:80. CSF IgM+ 1:10	C	[58]
2013, 3	Prodrome, HA, fever+, mosquitoes	0.9	CT, LP+,	HIV, WNV, Lyme, CMV, HSV, VZV	0.9	SSHV	NR	Serum JCV IgM+ in both samples, PRNT 0 to 1:40 later	C	[58]
2016, 5	Fever+, rash,	0.9	CT, MRI	HIV, Parvo B19, WNV POW, Lyme	NR	SSHV IgM+, PRNT 1:40 to 1:80	NR	Serum 1:320 to 1:1280, CSF IgM+ 1:4	C	[58]
2011 TO 2016	Confusion+, ataxia+	NR	NR	EEE, POW, SLE, but WNV IgM+	NR	LACV	NR	IgM + and PRNT confirmed JCV	C	[12]
2013, 1	Prodrome, rash, chills	0.4	LP+, MRI+,	WNV, EEE, Lyme, Babesia microti (IgM+)	<1.0	LAC	NR	Serum IgM+, PRNT 1:160	P4	[50]
2014, 2	Long pro-drome, HA, confused+, left hand numb+	3.0	LP+, MRI+, brain biopsy	Serum WNV, EEE, POW and CSF WNV IgM, CSF EEE IgM	3.0	LAC	NR	IgM+, PRNT 1:1280, both in CSF	P4	[50]
2014, 3	Long pro-drome, HA, confused	No LP	None	WNV, SLE, EEE, POW	10.0	LACVPRNT+ at 1:160	NR	IgM+, PRNT 1:1280 in serum	P4	[50]
2014, 4	Prodrome, fever+, HA	No LP	None	WNV, EEE	0.4	LACVPRNT+ at 1:160	NR	IgM+, PRNT 1:320 in serum	P4	[50]
2015, 5	Prodrome, HA, paresthesia	3.0	LP+, MRI+	WNV, EEE, POW, and WNV	3.0	LACV1:20	NR	IgM+, PRNT 1:160	P4	[50]
2016,6	Long pro-drome, HA, fatigue, myalgias	NR	LP, MRI.	WNV, EEE, POW, but Lyme IgM and Western blot IgG+	NR	NR	NR	IgM+, PRNT 1:80	P1, P4	[50]
2016, 7	HA, fever+, confused+	0.9	LP+, MRI	WNV, POW	<1.0	LACV1:40	NR	IgM+, PRNT 1:2560	P4	[50]
2017, 8	HA, confused,	2.5	LP, CT, MRI	WNV, POW, POW- in CSF	2.5	LAC	NR	IgM+, PRNT 1:160	P4	[50]
2017, 9	HA, confused+, seizure+	0.1	LP, MRI	WNV, EEE, POW	<1.0	LAC	NR	IgM+, PRNT 1:160	P4	[50]
2014	Prodrome, HA, fever, sepsis, rash mosquitoes, stiff neck	No LP	CT pelvis and abdomen	19 serological tests	3.9	LACV IgM negative, PRNT <1:10 to 1:320	CSG	JTC IgM negative, PRNT 1:160 rose to 1:10,240	P4	[60]
2015	HA, fever+, confusion+	0.7	LP, MRI, EEG	HSV, Bart, Borr, Coxac, Anaplas, autoimmune	4.1	IgM+, SSH rose 1:20 to 1:320	CSG 4.1	IgM+, JTC rose 1:40 to 1:320	P4	[68]
2016	Prodrome, HA, seizure+	0.7	LP, MRI, EEG+, brain biopsy+	Extensive infectious, autoimmune	0.9	NR	4.0	Initial serology and later PRNT+	P3	[62]
2017	Complex migraine-like HAs twice, also papilledema	0.2	LP+, CT, CTA, EEG, MRI+	>15 micro-biology tests	0.2	SSH IgM+ serum and CSF, PRNT negative serum	NR	IgM+ serum and CSF, and PRNT+ > 1:80 in serum	C	[67]
2017	Prodrome, HA, fever+, Confused+, known liver transplant	0.9	LP+, auto- immune Ab panel both CSF serum, MRI	>15 micro-biology tests, anti-body panel for viruses, mMGS	0.9	LACV1:10	8.0	CSF IgM+. PRNT in CSF 2 and 1:320 serum	C	[46]
2017	11-month prodrome, then rapid dementia	NR	LP+, MRI, PET, EEG+, brain biopsy+	Auto-immune panel, >15 microbio-logy tests	NR	LAC	NR	RT-PCR+, mNGS positive	C	[4]
2018	Prodrome, HA, ataxia+, diplopia, mosquitoes	0.1	LP+, CT, MRI+, EEG	>15 micro-biology tests	0.1	LACV 1:20, SSH 1:20 both stable in later sera	NR	IgM+ and PRNT 1:2560 in CSF and serum	C	[56]
2018	Confusion+, Hallucinations, known heart transplant. Admission day 10	1.4	LP+, CT, MRI, EEG, cardiac biopsies	Autoimmune panel, >15 micro-biology tests, CSF encephalitis panel	5.1	LACV	6.6	RT-PCR negative throughout, IgM+ serum and CSF, PRNT rose 2-fold in serum. IgM+ >7 months	C	[34]
2020	Dysarthria+, Confused+, mosquitoes	0.9	LP+, MRI+	CSF BioFire array for VZV+, but 13 agents negative	0.9	LACV IgM and ELISA	20.0	CSF IgM+ and PRNT 1:512 in serum	C	[66]
2021	Hallucinations+, HA, confusion+, mosquitoes, rash	0.4	LP+, CT, MRI+	CSG, WNV, SLE, EEE, WEE, LCEV, VDRL	0.7	NR	NR	IgM in serum and CSF, PRNT 1:160	P3	[52]
2022	L arm cramp+, aphasia	0.6	LP+, CT, CTA, MRI+, EEG+	Auto-immune, meningitis and tick panels, brain biopsy	1.6	LACV + 1:1 serum, CSF negative	NR	CSF at biopsy site: JTC IgM+ and PRNT > 1:64	C	[59]
2022	HA, diplopia+, confusion+	0.1	LP+, CT, MRI+, EEG+	CSF BioFire array, auto-immune panel	1.3	NR	>5.4	JTV IgM+, PRNT 1:40 in serum	C	[32]
2022	HA, fever+, stiff neck	0.1	LP, MRI+, meningitis panel	autoimmune and micro panels	0.1	LACV	3.9	JTCV IgM+ and PRNT+ in CSF	C	[72]
2022	seizure+	0.1	LP, MRI+, CSF shunt	Brain biopsy, low level viremia from CMV and EBV	NR	NR, but LACV unlikely given where she lived.	NR	CSF JTC RT-PCR+, Serum JTC IgM+, PRNT 1:40	C	[61]
2022	Fever+, confusion+	0.7	LP+, MRI+	CSF POW IgM+, PRNT 2, serum IgM+, PRNT 1:160	0.7	NR	5.0	CSF JCV IgM +, PRNT 8, serum IgM+, PRNT 1:640	C	[49]

(a) Year of infection onset, numbered cases in case series. (b) Reported clinical symptoms. HA = headache. Prodrome = patient-reported symptoms that may or may not be relevant to later diagnosis. + if diagnosis was bolstered. (c) Lumbar puncture delay from after first clinical contact with author(s) of report. Day 1 of illness = authors’ estimates of the time of disease onset or date of first clinician contact or documented fever, new severe HA, new seizure, or other objective findings. NR if reported data insufficient; (d) Reported testing for possible infections. + if diagnosis was bolstered; F: complement fixation; CT = computed tomography scan of brain; CTA: Computed Tomography Angiography; IF = immuno-fluorescence; IgM = MAC-ELISA (antigen-capture enzyme-linked immunosorbent assay) preceded by virus abbreviation if virus-specific; LP = lumbar puncture; MRI = magnetic resonance imaging of brain; PRNT = plaque reduction neutralization test; RT-PCR = reverse-transcriptase-polymerase chain reaction; NR = not reported. (e) Serology tests other than for CSG viruses, + if positive; AFB: acid-fast bacilli; CMV: cytomegalovirus; EBV: Epstein–Barr virus; EEEV: Eastern encephalitis virus; HBV: hepatitis B virus; HCV: hepatitis C virus; HHV6: human herpes virus 6; HIV: human immunodeficiency virus; HSV1: herpes simplex virus; meas: measles; MOGAD: myelin oligodendrocyte glycoprotein antibody-associated disease; mmp: mumps; NMDA: N-methyl-D-aspartate receptor; POW: Powassan virus; RMSF: Rocky Mountain spotted fever; RSV: respiratory syncytial virus; SLE: St. Louis encephalitis virus; VDRL: syphilis serology; VZV: varicella zoster virus; WEE: Western encephalitis virus; WNV: West Nile virus. (f) First clinical sample sent out that later made JCV etiology probable or confirmed, (g) CSG viruses tested for cross-reactivity with JTCV, + if positive, CEV = California encephalitis virus; JCV = Jamestown Canyon virus; KEYV = Keystone virus; LACV = La Crosse virus; SAV = San Angelo virus; SSHV = snowshoe hare virus; TAHV = Tahyna virus; TVTV = Trivitattus virus. HI: hemagglutination-inhibition; (h) weeks elapsed from JCV diagnostic sendoff until report sent to clinicians; (i) details of JCV results (j) Confirmed Reports (CR). Each case was analyzed to determine whether it was confirmed as a JCV case by CDC definition [59]. Probable cases were considered probable and not confirmed for one of four reasons: P1 = inadequate clinical detail for neuroinvasive and non-neuroinvasive disease or to exclude a more likely explanation of symptoms or no specification of type of clinician; P2 = JCV IgM positive, but PRNT unchanging; P3 = inadequate testing for other probable viral causes; P4 = inadequate testing by PRNT or IgM to exclude probable viral cross-reactive antibodies.

**Table 3:** JCV cases with complexity of being immune compromised or having other infections.

Year, Case ^a^	Other Infectious Agents ^b^	Clinical Notes ^c^	Laboratory and Serology Results ^d^	Notes ^e^	Ref
1980	HSV	Encephalitis, recurrent HSV	JTCV PRNT 4-fold rise; HSV 16-fold rise	HSV not detected in CSF Recovered	[24]
1982, 10	HSV	Encephalitis	JTCV PRNT, HI stable but IgM positive	HSV encephalitis? Death	[10]
1997	HSV?	Prolonged encephalitis	Slow 2-fold JCV PRNT rise. Low HSV titers	RT-PCR positive for JCV in CSF and brain biopsy, disabled	[26]
2011–2016	WNV	Encephalitis	PRNT confirmed both viruses recent	Limited details of case Death	[12]
2013, 1	*Borrelia burgdorferi*	Encephalitis	Lyme IgM Western blot+ JCV IgM+ with PRNT 1:160	Poor memory, ataxia, depression	[50]
2015	SSHV	Encephalitis,	JTCV IgM+ and 8-fold PRNT rise, SSHV IgM+ and 16-fold PRNT rise	Cross-reactivity of viruses, long term symptoms	[68]
2016, 7	*Borrelia burgdorferi*	Rheumatoid arthritis (prednisone 10 mg daily).	Engorged tick JTCV IgM+ with PRNT 1:2560	Empirical Lyme treatment, previously on methotrexate Ongoing fatigue	[50]
2017–2018	-	Rituximab for lymphoma,	RT-PCR and mNGS positive at end of life	One year of progressive dementia Death	[4]
2018	EBV	Heart transplant on tacrolimus and mycophenolate, encephalitis	Serum JCV PRNT rose only 2-fold, but serum and CSF IgM favored JCV over EBV. RT-PCR for JCV was positive at 7.5 weeks	Recovery after immune reconstitution and ribavirin Recovered	[34]
2018–2023	EBV	TYK2 deficient, pneumonia, encephalitis	CSF JCV IgM+ with PRNT 1:40	Very complex case Long term neurological impairment	[61]
2019	VZV	Encephalitis, L5 dermatome rash,	CSF: VZV PCR+ and JCV IgM and PRNT 1:512	Death	[66]
2017	-	Liver transplant on cyclosporin and prednisone, encephalitis	CSF IgM and serum PRNT 1:320	Treated with IV IgG Recovered	[46]
2022	*Borrelia burgdorferi*	Erythema migrans and meningitis	Lyme serum IgM+, JCV CSF IgM and PRNT+	Briefly hospitalized for antibiotics, recovered	[72]
2022–2023	POW	Encephalitis	CSF IgM and PRNT positive for POWV and JTCV	RT-PCR and mNGS negative for both viruses, recovered	[49]

(a) Year of infection onset, numbered cases in case series. (b) Identified potential co-infection or previous infective agent. EBV: Epstein–Barr virus; HSV: herpes simplex virus; POW: Powassan virus; SSHV = snowshoe hare virus; VZV: varicella zoster virus; WNV: West Nile virus. (c) Notable clinical notes. (d) Serology test results. (e) Notes on case.

### 3.5. Outcomes of JCV Encephalitis

We next evaluated the outcomes of JCV cases. Deaths occurred in 10 (2.4%) of the original 416 cases that included a diagnosis (Table S1). All deaths were associated with encephalitis, with death occurring in 6.5% of the 154 encephalitis cases. Of the detailed 49 case reports, 40 had clear reports of outcome (Table 1). Of these, there were four reported deaths related to JCV [4,10,12,66] and one death related to sepsis [62]. Prolonged impairment or recovery time of two months or more after JCV encephalitis was reported for 16 cases [26,32,49,50,52,58,61,68]. However, there were also 19 cases with short hospital stays and full recoveries within two months [10,42,55,56,58,67]. Thus, approximately half of the cases with clear reported outcomes had long-term consequences of JCV infection.

### 3.6. Confounding Factors of JCV Cases

We analyzed individual JCV cases for potential confounding factors that could have impacted the diagnosis, treatment, or outcome, including immune status and previous or co-infections (Table 3). Of the 49 case reports, five (10%) patients were immunocompromised, which included inherited immune deficiency, prednisone treatment of rheumatoid arthritis, rituximab therapy for lymphoma, or immunosuppression to prevent transplant rejection [4,34,46,50,61]. Analysis of the 49 detailed cases, as well as a report of 30 cases where enhanced surveillance for other pathogens was completed [12], identified positive tests or NAb titers for other infectious agents in 16 of 79 cases (19%). These agents included herpes simplex virus 1 (HSV1), varicella zoster virus (VZV), Epstein–Barr virus (EBV), SSHV, WNV, Powassan virus (POWV), and *Borrelia burgdorferi* (Table 3). Not surprisingly, three of the immunocompromised cases had evidence of other infections besides JCV [34,50,61]. Of the 14 cases from Table 1 that had confounding factors of immunosuppression or pre/co-infection, the case outcomes were four deaths, four long-term neurological sequelae, and six recoveries. Thus, the rates of severe outcomes were higher in the cases with confounding factors but without a clear correlation with being immunocompromised or with any specific additional pathogen. However, confounding factors do appear to potentially have a negative impact on the outcome of JCV-related disease.

## 4. Discussion

In this study, we analyzed the reported cases of JCV over 40 years, from 1982 to 2022. Prolonged hospital stays and long-term impairment or recovery were commonly observed, with approximately 40% of cases reporting symptoms lasting 2 months or more. These outcomes correlate with comprehensive reviews of outcomes and longer-term impairment from other encephalitis viruses commonly found in North America, with discharged patients frequently having deficits in cognitive function such as memory loss or attention disorders [73]. For example, long-term symptoms are observed in 30–70% of patients with HSV-1 encephalitis, 30–50% of patients with varicella zoster virus (VZV), and up to 75% of patients with WNV neurological disease [73]. For patients hospitalized with LACV, 12% had clear neurological deficits at discharge, and 22% had cognitive and/or behavioral deficits at 10–18 months post release [74]. Long-term assessments of hospitalized children with LACV showed long-term neurobehavioral deficits in 19–54% of patients [75]. A direct comparison of JCV cases with these causes of viral encephalitis is difficult due to the low number of cases with JCV, as well as the differences in age between JCV and LACV patients. However, in all encephalitic viruses examined, at least one-third of cases reported impairment of over 2 months due to viral encephalitis. Thus, although many viruses such as JCV have low rates of mortality, they do consistently cause long-term consequences that can strongly impact quality of life. Better diagnosis and therapeutics that prevent neurological damage could be critical in increasing quality of life in these patients.

One of the surprising findings in this analysis was that the time from clinical onset to testing for JCV has not decreased over time (Figure 2). However, to recognize JCV infection early in the disease process, a clinician needs to recognize the acute encephalitis syndrome (AES) and have access to clinical laboratories that perform rapid, comprehensive analysis of serum and CSF for JCV. There may be many reasons why this window of time has not improved, but one reason may be the lack of specific, not just supportive, therapy, which makes a clear diagnosis less beneficial for the patient. Indeed, effective treatment for herpes simplex virus fostered more rapid specific diagnosis [69,76,77]. Thus, therapeutic breakthroughs for the treatment of encephalitic viruses such as JCV may not only improve the outcome of these patients but also reduce the percentage of viral encephalitis of unknown causes.

Another reason for the lack of improvement in time from clinical onset to diagnosis may be the diagnostic testing itself. Serological analysis of IgM and subsequent PRNT has remained the primary diagnostic test, with an increase in PRNT needed for confirmation. This is often difficult, as the results are received after the patient is discharged. RT-PCR testing could speed diagnosis and avoid problems of cross-reactivity of JCV [26]; however, RT-PCR does not always detect active infections [4,27,34,49,61]. Indeed, RT-PCR testing is currently recommended by the CDC only for patients who are immunocompromised, as these patients may have persistent viremia. However, routine analysis for JCV by the CDC JCV IgM ELISA has increased the number of cases [12], indicating that better diagnostics impact case detection. Further improvement of diagnostic tests and more widespread use may provide a more accurate understanding of JCV and other arbovirus cases in North America.

Due to the limited diagnostic tools over the last 40-year span, JCV cases are undoubtedly underreported, which can impact our understanding of the impact of individual viruses [78]. This may be truer for non-neurological JCV cases than neurological JCV cases. In an enhanced surveillance study with direct comparison of 15 neuroinvasive and 15 non-neuroinvasive JCV cases, the investigators found few early characteristics that differed between the neuroinvasive and non-neuroinvasive groups [12]. County-by-county maps of acute human JCV infections are based on data collected only since 2011 and thus cannot be correlated with historical infections. Surveillance funding for JCV cases was discontinued in the mid-1980s in upstate New York, which demonstrates the lack of information to estimate the distribution of current infections. Additionally, reported cases of JCV are often dependent on samples being sent by clinicians to state or federal labs for analysis [28,36,40,45,79,80,81,82,83], which can vary depending on funding for testing, awareness, and reporting criteria. Finally, the 49 detailed cases reported in the literature may have confounding factors that lead to more detailed reporting and may not be representative of average cases. Under-ascertainment and underreporting of infectious diseases can limit our understanding of the consequences of different pathogens on human disease [78]. Thus, the full impact of JCV infection on human health is underestimated and in need of further clinical attention.

## Data Availability

All data are listed or cited in the manuscript.

## Supplementary Materials

The following supporting information can be downloaded at: https://www.mdpi.com/article/10.3390/v18020271/s1.
