# Supplementary material for: Jamestown Canyon Virus Disease: An Analytic Review of Human Cases Reported from 1982 Through 2022
Source: Viruses. 2026 Feb 23;18(2):271. doi: 10.3390/v18020271 (PMC12945000; doi:10.3390/v18020271)
Supplement: Supplementary file 1 [file viruses-18-00271-s001.zip › viruses-4066349-supplementary.pdf]

Table S1. Reports of Human Acute Jamestown Canyon Virus Infections 1971-2022

Case years[a]	Print year[b]	Infections & location [c]	Report type [d]	Diagnoses reported [e]	Ref.
**1980**	1982	1 MI	CR	1 AES	(22)
**1980-1982**	1983	1 MI, 10 NY, 1 ON	CS	3 AES, 4 M, 1 NI, 3 NN, 1 NR; 1 death	(10)
**1971-1981**	1984	41 NY	Lab	1 E, 7 M, 33 NR	(25)
**1982**	1984	23 NY	Lab	3 E, 14 M, 4 NI, 2 NN	(25)
**1982-1983**	1986	2 IL, 1 OH	Lab	1 AES, 1 M, 1 NN	(22)
**1984**	1985	1 NT	CNML	1 M	(33)
**1986**	1987	2 IN	CDC	2 NR	(38)
**1997**	1999	1 NY	CR	1 AES	(26)
**2000-2013**	2015	1 CA, 1 CT, 1 ID, 1 MA, 2 MN, 3 MS, 1 MT, 1 NH, 4 NY, 1 OR, 1 PA, 1 RI, 13 WI	CDC, *ArboNET*	11 ME, 6 M, 7 NN, 7 NR	(27)
**2001**	2002	1 CT	Lab	1 M	(72)
**2004**	2004	1 WI	*ArboNET*		
**2006**	2006	1 MS	*ArboNET*		
**2008**	2008	1 MS, 2 NY, 1 WI	*ArboNET*		
**2009**	2011	1 MT	CR	1 E	(42)
**2011**	2011	1 MS, 2 WI	*ArboNET*	2 NI, 1 NN	
**2011-2016**	2024	2 AB, 1 BC, 1 NS, 1 QC	CS	4 E, 1 M	(58)
**2012**	2012	1 NY, 1 WI	*ArboNET*	1 NI, 1 NN	
**2011-2016**	2019	30 WI	Lab	6 ME, 9 M, 15 NN	(12)
**2013-2017**	2020	9 MA	CS	4 AES, 2 E, 1 M, 2 NN	(50)
**2013**	2014	1 GA, 1 ID, 1 MA, 1 MN, 1 NH, 3 NY, 1 OR, 1 PA, 1 RI, 7 WI, 4 NR	CDC, *ArboNET*	9 E, 6 M, 7 NN	(54)
**2014**	2015	1 MA, 4 MN, 1 TN, 5 WI	CDC, *ArboNET*	3 E, 2 M, 1 AFP, 5 NN	(53)
**2014**	2015	1 MN	CR	1 E	(60)
**2015**	2017	1 NB	CR	1 E	(68)
**2015**	2017	1 IA, 1 MA, 1 MN, 1 NJ, 1 OH, 2 WI, 1 WY, 3 NR	CDC, *ArboNET*	4 E, 1 M, 1 AFP, 1 NI, 5 NN	(51)
**2015**	2016	30 Canada	CNML	30 NR	(79)
**2016**	2017	14 Canada	CNML	14 NR	(80)
**2016**	2018	1 QC	CR	1 AES; 1 death	(62)
**2016**	2018	1 MA, 1 MN, 5 WI, 8 NR	CDC	4 E, 2 M, 1 NI, 8 NN	(35)
**2017**	2018	1 LA, 2 MA, 2 ME, 22 MN, 1 NC, 3 NH, 1 OH, 43 WI	CDC *ArboNET*	29 E, 5 M, 4 AFP, 20 NI, 17 NN; 2 deaths	(47)
**2017**	2018	89 Canada	CNML	89 NR	(81)
**2017**	2018	1 MB	CR	1 ME	(67)
**2017**	2022	1 NC	CR	1 AES	(46)

**2017-2019**	2022	3 ME	ME Public Health	2 NI, 1 NN; 1 death	(82)
**2018**	2018	1 MN	CR	1 ME	(56)
**2018**	2018	48 Canada	CNML	48 NR	(83)
**2018**	2019	1 CT, 1 MA, 1 ME, 1 MI, 7 MN, 1 TN, 13 WI, 16 NR	CDC	11 E, 7 M, 4 AFP, 3 NI, 16 NN; 1 death	(57)
**2018**	2019	1 WI	CR	1 E	(34)
**2018**	2021	1 MA	CR	1 AES; 1 death	(4)
**2019**	2021	1 IL, 3 MA, 1 MI, 11 MN, 3 NH, 6 WI, 20 NR	CDC	14 E, 4 M, 1 AFP, 6 NI, 20 NN; 2 deaths	(65)
**2019**	2022	2 QC, 1 NT	CNML	3 NR,	(28)
**2020**	2020	1 MI	CR	1 AES; 1 death	(66)
**2020**	2022	3 CT, 3 MI, 3 NH, 4 WI	CDC	9 E, 1 NI, 3 NN	(63)
**2021**	2021	1 MI	CR	1 AES	(52)
**2021**	2022	1 MI	CR	1 AES	(59)
**2021**	2022	1 RI	CR.	1 AES	(32)
**2021**	2023	1 IN, 1 ME, 6 MI, 6 MN, 1 RI, 5 NH, 2 NJ, 10 WI	CDC	13 E, 4 M, 1 AFP, 3 NI, 11 NN	(48)
**2022**	2023	1 WI	CR	1 M	(69)
**2022**	2023	1 NT	CR	1 AES	(61)
**2022**	2023	1 NJ	CR	1 AES	(49)
**2022**	2024	1 MA, 3 MI, 1 MN, 2 RI, 4 WI, 1 NR	CDC	6 E, 3 M, 2 NI, 1 NN	(64)
**1971-2022**	**Total cases**	598 **Total in diagnostic categories**	416	AES = 19, E = 116, ME = 19, M = 78, AFP = 12, NI = 47, NN = 125, NR = 182. All 10 deaths were associated with encephalitis.	

(a) Listing by year of the first acute case during the study interval.

(b) Year that the study was published.

(c) number of cases and state/providence cases occurred

(d) where information of case was obtained: CR = case report, CS = case series, Lab = laboratory-sponsored case report or case series. *ArboNET* = online access to preliminary case numbers and locations reported to the CDC.

(e) Diagnosis of disease: AES = acute encephalitis syndrome criteria (2). AFP = acute flaccid paralysis, E = encephalitis, M = meningitis, ME = meningoencephalitis, NI = neuroinvasive, NN = not neuroinvasive, NR = not reported.
